# Significance of anti‐desmocollin autoantibodies in pemphigus

**DOI:** 10.1111/1346-8138.16660

**Published:** 2022-12-28

**Authors:** Norito Ishii

**Affiliations:** ^1^ Department of Dermatology Kurume University School of Medicine Kurume Japan

**Keywords:** autoimmune bullous diseases, desmocollin, desmoglein, desmosomes, pemphigus

## Abstract

The major autoantigens for pemphigus are desmogleins (Dsgs), cell–cell adhesive structure proteins, one of the desmosomal cadherins. Recent progress in molecular biology has revealed that IgG autoantibodies of classical pemphigus react with Dsg1 or Dsg3. Desmocollins (Dscs) also belong to the cadherin supergene family that provides structure to the desmosomes and play an important role in cell‐to‐cell adhesion. In addition to the presence of four desmosomal Dsg isoforms, i.e. Dsg1‐4, Dsc1, 2 and 3, all of which are derived from different genes, Dsc1 has been previously identified as the target antigen of IgA autoantibodies in the subcorneal pustular dermatosis (SPD)‐type of intercellular IgA dermatosis. In addition to the IgA anti‐Dsc1 autoantiboides, the presence of IgG anti‐Dsc autoantibodies is described in patients of some autoimmune bullous diseases. In particular, the current pemphigus detecting autoantibodies to Dscs has shown a tendency in atypical variants of pemphigus. Therefore, autoantibodies against Dscs alone may cause detachment of cell–cell adhesion in the epidermis in some pemphigus. However, except for the findings of a few in vitro and in vivo studies, there is currently no clear evidence for the pathogenicity of anti‐Dsc autoantibodies in pemphigus, whereas significance of anti‐Dsg autoantibodies is well established. This article describes the structure and function of the Dscs, and explores the evidence regarding the pathogenic role of anti‐Dsc autoantibodies in pemphigus.

## 1 INTRODUCTION

To form the human body and maintain the integrity of its complex tissues, individual cells need to bind tightly to each other. There are two major cell adhesion structures, i.e., desmosomes for adhesion between keratinocytes and hemidesmosomes for adhesion between the epidermis and the underlying dermis. Autoimmune bullous diseases (AIBDs) encompass a group of disorders characterized clinically by blisters and erosions in the skin and/or mucous membranes. AIBDs, such as pemphigus and pemphigoid, are mediated by essentially IgG autoantibodies against different structural proteins of these desmosomes and hemidesmosomes, respectively.^1^

Pemphigus is a group of AIBD characterized by IgG production against desmogleins (Dsgs), and is divided into two major classic forms; i.e., pemphigus foliaceus (PF) and pemphigus vulgaris (PV).^2^, ^3^ The well‐known major autoantigens for classical pemphigus are Dsg1 and Dsg3.^4^ Furthermore, desmocollin (Dsc)1 was identified as an autoantigen in subcorneal pustular dermatosis (SPD)‐type intercellular IgA dermatosis (also known as IgA pemphigus) by cDNA transfection method.^5^ Using the same detection method, IgG anti‐Dsc autoantibodies were detected in some patients with atypical pemphigus.^6^, ^7^, ^8^, ^9^

Despite the established pathogenic role of anti‐Dsg autoantibodies in classical pemphigus, the significance of anti‐Dsc autoantibodies is largely unknown. This article outlines the structure and functional characteristics of Dscs, and explores the evidence regarding the pathogenic role of anti‐Dsc autoantibodies and provides insights on its association with clinical features of pemphigus.

## 2 MOLECULAR STRUCTURE AND PHYSIOLOGICAL FUNCTION OF DSC

Dsg and Dsc, two representative cadherin proteins that play important roles in cell–cell adhesion are located in desmosomes. In human genomes, there are three isoforms of Dscs 1–3 and four isoforms of Dsgs 1–4,^10^ which share an overall domain organization comprising four to five extracellular cadherin (EC) domains, a single‐pass transmembrane region, and an intracellular domain that binds to intermediate filaments via adaptor proteins desmoplakin and plakoglobin.^11^

**FIGURE 1:**
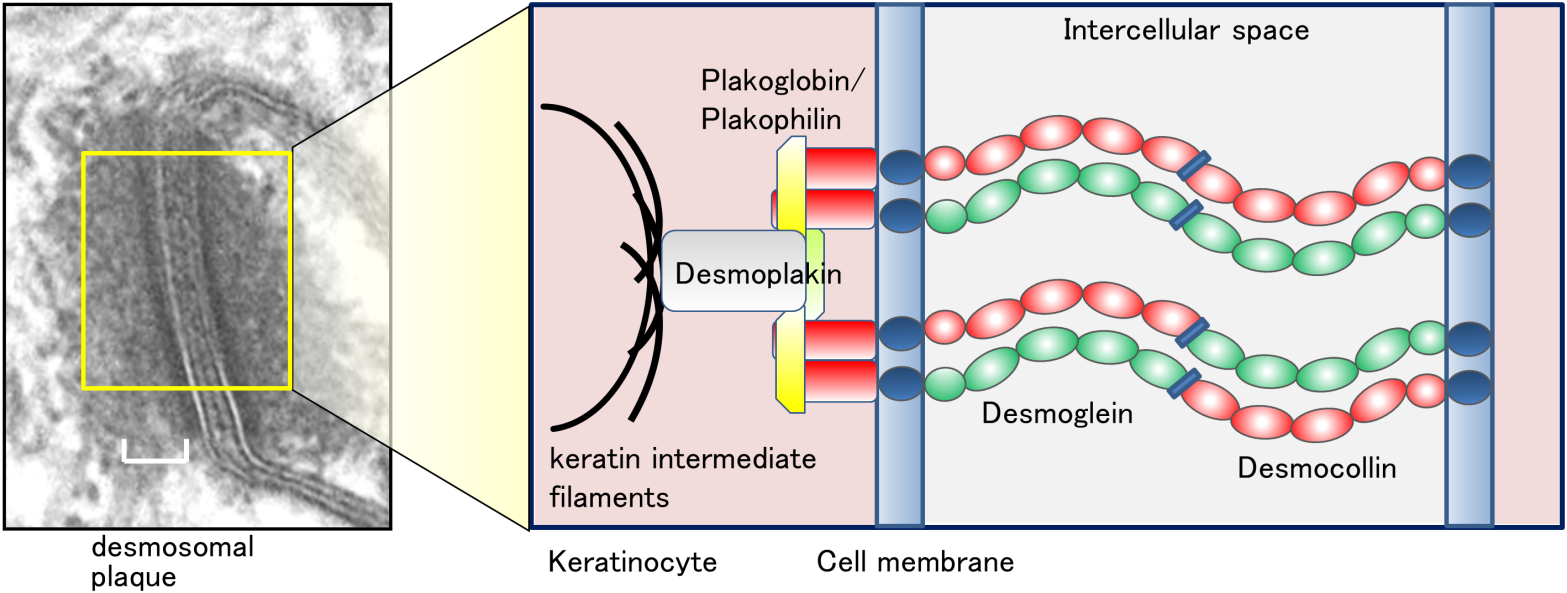
Electron microscopic view of a desmosome and schematic representation of desmosomal proteins. Intercellular adhesion of desmosomes is mediated by homophilic and heterophilic binding of the desmosomal transmembrane proteins, desmogleins and desmocollins. The cytoplasmic domains of these proteins are bound to keratin intermediate filaments via plakoglobin, plakophilin, and desmoplakin.

**FIGURE 2:**
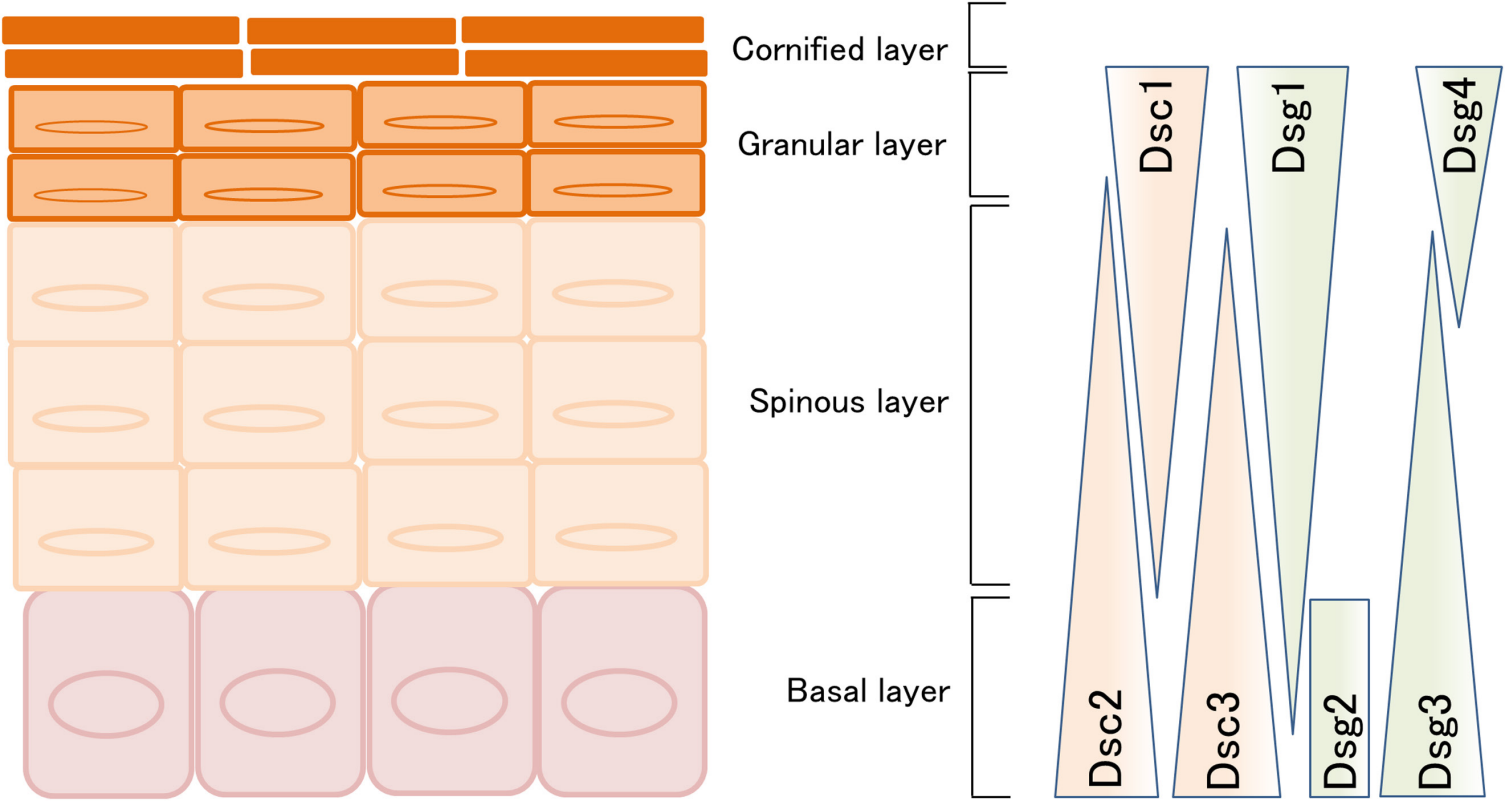
Desmosomal cadherin expression patterns of cutaneous epithelia.

All three Dsc gene products undergo alternative splicing, resulting in the generation of the Dsc a larger “a”‐form and a shorter Dsc “b” form of the proteins, which differ in the length of their respective carboxy‐terminal domains.^12^, ^13^ Dsc “a” isoform and Dsgs contain an intracellular cadherin‐like sequence (ICS) that binds plakoglobin. On the cell surface, Dsc and Dsg bind to each other, providing support, and meditating connections between intermediate filaments of neighboring cells.^14^, ^15^ In addition, this cadherin complex forms an anchor for keratin intermediate filaments which attach to the inner cytoplasmic surface (Figure 1).^16^ Individual Dsgs and Dscs show differential expression patterns. Various desmosomal cadherins are expressed specifically in stratified epithelia and exhibit a stepwise overlapping pattern (Figure 2).^17^ Dsg2 and Dsg3 are mainly distributed throughout the lower layers of the epidermis, whereas Dsg1 is expressed at higher levels in the upper layers. Dsg4 is primarily expressed in the hair follicle and in the granular layer. Dsc2 and Dsc3 are present in the basal and spinous layers, whereas Dsc1 is expressed in the granular layer. Notably, both Dscs and Dsgs appear necessary for adhesion in epithelial cells,^18^, ^19^, ^20^ and loss of either in genetic experiments causes loss of normal desmosomal adhesion.^21^, ^22^, ^23^

According to the ‘Dsg compensation theory’, the autoantibody profile critically affects the clinical features and the histological site of blistering.^24^ PV is characterized by suprabasal loss of adhesion of the skin and oral mucous membrane, where Dsg3 is predominantly expressed. In contrast, PF is characterized by superficial blisters of the skin but not of the mucosa, since Dsg1 is preferentially expressed in the subcorneal epidermis of the stratified epithelia. Moreover, the clinical phenotype of each pemphigus variant can be largely explained by the tissue expression pattern of these autoantigens.^24^, ^25^

The role of Dsc on the cell–cell adhesion in keratinocytes is expected by the study of Dsc gene targeting mice. Mice lacking Dsc1 (Dsc1−/−) showed epidermal fragility resembling pemphigus, and defects of epidermal barrier function.^21^ Loss of Dsc3 in mice (Dsc3−/−) has also shown epidermal blistering.^22^ These previous knockout mice studies showed that Dsc expressions are necessary for normal cell adhesion in desmosomes.

Even more, there has been controversy as to whether desmosomal cadherins have a homophilic binding preferences of Dsg and Dsc or whether interactions occur in heterophilic pairs.^18^, ^26^ Previously, the desmosomal cadherins, Dsg and Dsc, are assumed to be homophilically binding, as are classical cadherins, but no direct proof has been shown. The possibility of heterophilic adhesion has also been pointed out. Recent studies showed that heterophilic interaction of Dsg and Dsc (Dsg/Dsc) was more dominant for cell adhesion over a homophilic interaction of Dsg or Dsc (Dsg/Dsg or Dsc/Dsc) by solution biophysical analysis and coated‐bead aggregation assays.^27^ Furthermore, using coated‐beads aggregation assays, data supporting the heterophilic theory showed that a pathogenic pemphigus mAb blocked heterophilic interaction of Dsg/Dsc without the involvement of intracellular signaling or other cellular processes, suggesting steric hindrance by the autoantibodies as the primary pathogenic mechanism in pemphigus.^28^, ^29^

## 3 IGA ANTI‐DSC AUTOANTIBODIES IN PEMPHIGUS

**FIGURE 3:**
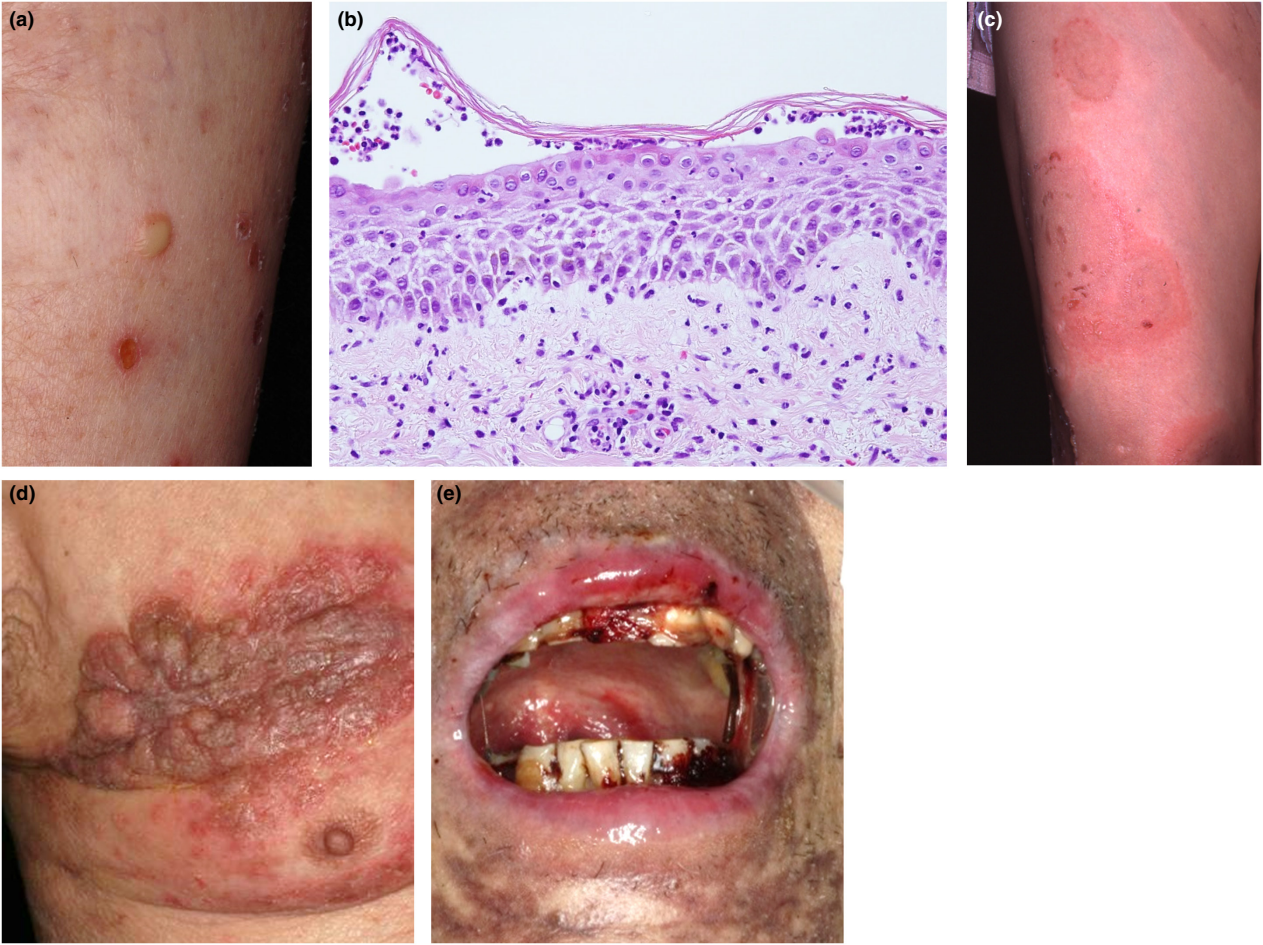
Clinical, histological features of pemphigus detecting each anti‐desmocollins autoantibodies. (a, b) Clinical features of subcorneal pustular dermatosis‐type of intercellular IgA dermatosis. Papulovesicles and subcorneal neutrophil pustules are observed (×200). (c) Clinical features of pemphigus herpetiformis. (d) Clinical features of pemphigus vegetans. (e) Clinical features of paraneoplastic pemphigus.

In general, the report associated with the pathogenic role of anti‐Dscs autoantibodies on AIBDs is limited. Among them, previously, IgA autoantibodies to Dsc1 were identified in SPD‐type of intercellular IgA dermatosis by cDNA transfection and living cell IF.^5^, ^30^ One of the subtypes of pemphigus, intercellular IgA dermatosis, is a rare AIBD characterized by the deposition of IgA autoantibodies at keratinocyte cell surfaces and is divided into two major subtypes, SPD‐type (Figure 3a,b) and intraepidermal neutrophilic dermatosis (IEN)‐type.^31^, ^32^ Dsc1 has been suggested to play an important role for the pathogenesis of SPD‐type, but the autoantigen for the IEN‐type has not been determined to date. Furthermore, using immunoelectron microscopic analyses, in SPD‐type, gold particles labeling the patient's sera were observed predominantly in the extracellular spaces between keratinocytes at desmosomes, and rarely in the intracellular domain at the desmosomal attachment plaques. In contrast, regarding the IEN type, they were observed in the intracellular spaces of nondesmosomal areas.^33^

Concomitant lymphoproliferative disorders and solid malignancies have been reported in intercellular IgA dermatosis, especially IgA monoclonal gammopathy of undetermined significance and IgA type multiple myeloma.^34^ For management of intercellular IgA dermatosis, daiphenyl sulfone (Dapsone) has proved to be the first line drug.^32^, ^35^ Dapsone generally inhibits polymorphonuclear leoukocyte cytotoxicity in neutrophilic dermatosis including intercellular IgA dermatosis.^32^, ^36^

## 4 DETECTION OF IGG ANTI‐DSC AUTOANTIBODIES IN PEMPHIGUS

From a clinical point of view, although a few cases of mucosal dominant‐type PV with exclusive anti‐Dsc3 autoantibodies have been reported, most of the anti‐Dsc3 autoantibody positive cases belong to atypical variants of pemphigus. Various reports of the presence of IgG anti‐Dsc autoantibodies have been found in classical or non‐classical types of pemphigus, such as paraneoplastic pemphigus (PNP), pemphigus herpetiformis (PH), and pemphigus vegetans (PVeg).^37^ PNP is an autoimmune multiorgan syndrome with severe polymorphous mucocutaneous manifestations associated with an underlying neoplasia (Figure 3e).^38^ PNP autoantibodies typically bind to multiple proteins; Dsg1, Dsg3, plakin family proteins, including desmoplakin I and II, bullous pemphigoid antigen I (BPAG1e), periplakin, envoplakin and alpha‐2‐macroglobulin‐like‐1 (A2Ml1).^39^, ^40^, ^41^, ^42^ In PNP, Dsc1, Dsc2 and Dsc3 were also variably found. Approximately 60% of PNP patients demonstrated autoantibodies against proteins of the Dsc family, including Dsc1, Dsc2 and Dsc3.^43^, ^44^ Particularly, anti‐Dsc3 and anti‐Dsc2 autoantibodies were frequently and specifically detected in PNP sera.^44^ PH is recognized as a distinct variant of pemphigus that is characterized by its pruritus, urticarial erythema with annular configuration, histologically eosinophilic spongiosis of epithelium,^45^ differing from clinical phenotype of the classic IgG‐mediated pemphigus (Figure 3c). Clinical and histopathological features of PH are unique findings, distinct from the classic pemphigus groups, although PH is immunopathologically pemphigus‐like IgG anti‐cell surface antibodies. Target antigens for PH are usually Dsg1 and less commonly Dsg3.^46^ Recently, several cases of PH without anti‐Dsg1 or anti‐Dsg3 autoantibodies have been reported, and some patients show reactivity against other antigens such as Dscs. PVeg is a rare variant of PV characterized by its hypertrophic vegetating lesions in the folds and mouth and by the presence of IgG auto‐Dsg3 autoantibodies (Figure 3d).^47^ Additionally, IgG and IgA autoantibodies against Dscs are also present in patients with PVeg.^48^ Approximately 30%–40% of PH and PVeg sera showed relatively strong reactivity with Dsc1‐3 in various patterns, respectively.^44^

## 5 DETECTION SYSTEM OF ANTI‐DSC AUTOANTIBODIES

Diagnosis techniques such as fluorescence antibody method, immunoblotting, and enzyme‐linked immunosorbent assay (ELISA) have been evolving at a rapid pace as tools for immunological diagnoses of AIBDs. They are getting to be absolutely essential for detections of antibodies, confirmed diagnoses, and decision‐making for therapeutic strategies. Various techniques were used to detect anti‐Dsc autoantibodies: immunoblotting, immunoprecipitation, indirect immunofluorescence with COS7 cells transfected with Dsc, and ELISA with baculovirus‐produced recombinant proteins or expressing human recombinant proteins in mammalian cells.

### 5.1 cDNA transfection method

**FIGURE 4:**
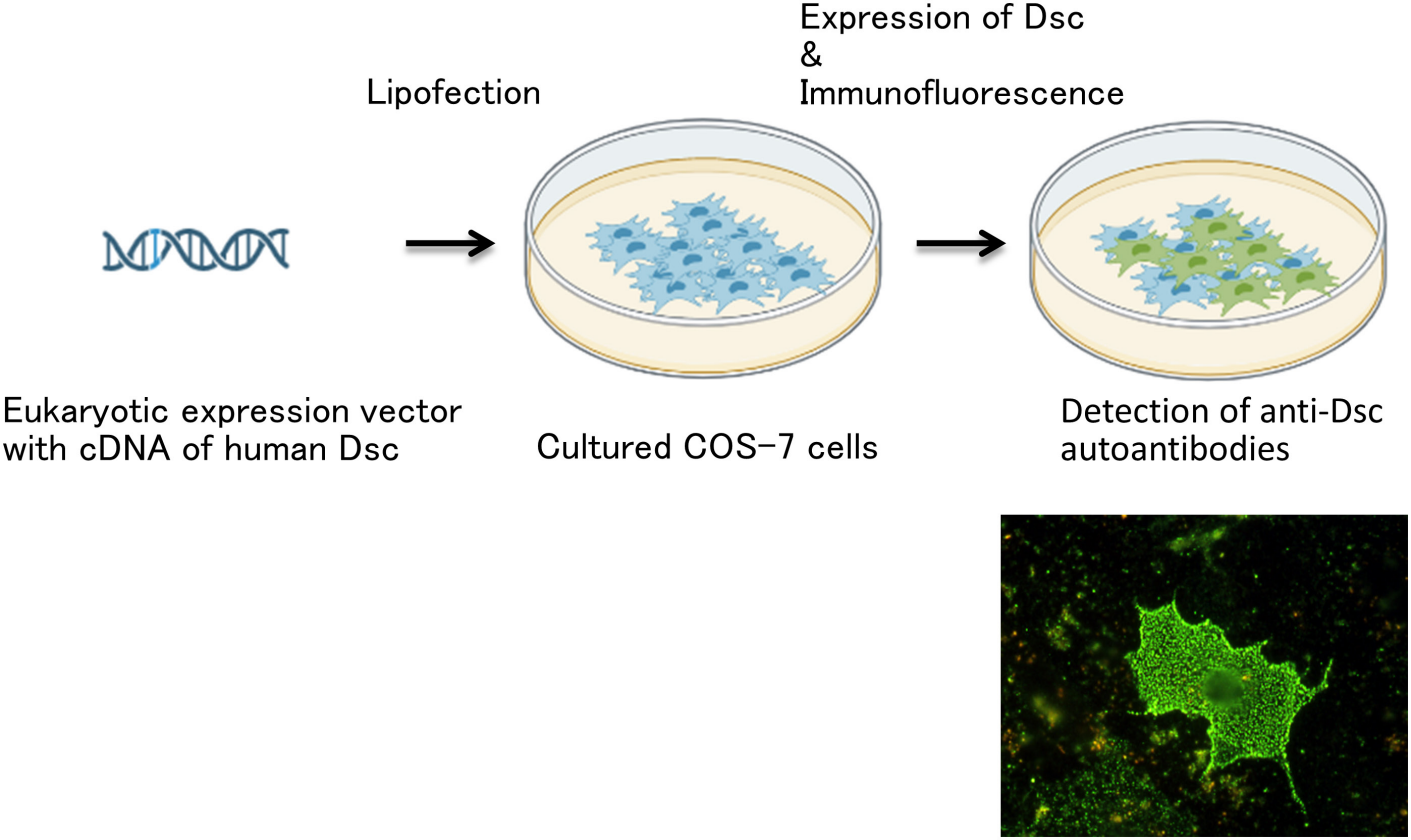
cDNA transfection method. Cultured COS7 cells transfected with cDNA vectors for human desmocollins (Dscs) are used as substrates for indirect immunofluorescence study to detect autoantibodies to Dscs. Positive reactivity of pemphigus with anti‐Dsc Abs to cell surfaces of COS7 cells transfected with cDNA of human Dscs.

Eukaryotic vectors containing full length complementary DNA (cDNA) of human Dsc1‐3 were transfected into cultured COS‐7 cells. IgA or IgG reactivity to each Dsc expressed on COS‐7 cell surface was detected by living cell immunofluorescence. Then, patient sera are reacted with these transfected cells. Dotted fluorescent signals are obtained at the cell surfaces if patient sera react with such target antigens (Figure 4). By this method, Dsc1 was identified as an autoantigen in SPD‐type intercellular IgA dermatosis. This cDNA transfection method is only available in special facilities, and is not widely available, mainly because of the difficulties in procedures and assessment.

### 5.2 Enzyme‐linked immunosorbent assay

First, ELISA using Dsc1‐3 recombinant proteins (RPs) produced in baculovirus system were developed. However, their sensitivities were not sufficient; ELISAs using baculovirus‐produced RPs failed to detect anti‐Dsc autoantibodies, even IgA anti‐Dsc1 autoantibodies in SPD‐type intercellular IgA dermatosis.^49^ The specificity and sensitivity of its ELISA is not very high, if compared to cDNA transfection method. Post‐translational modifications in ELISA were not probably suitable that in the recombinant proteins expressed in *Escherichia coli* or insect cells, it may be difficult to detect antigen–antibody reactions. Comparison of ELISA reactivity on plates prepared with recombinant Dscs protein expressed in insect cells and animal cells showed better reactivity with the recombinant protein in animal cells.^44^ Among its improvements, ELISA using eukaryotic recombinant Dsc proteins established, which showed results consistent with those by cDNA transfection method. Then, we established more sensitive ELISAs using Dsc1‐3 RPs produced in mammalian expression system that detect IgG anti‐Dsc autoantibodies frequently in atypical variants of pemphigus such as PNP, PH, and PVeg, but rarely in classical pemphigus. IgA Dscs ELISAs were also established that successfully detected IgA anti‐Dsc1 autoantibodies in SPD‐type intercellular IgA dermatosis.^50^ These ELISAs for the specific detection of anti‐Dsc autoantibodies are not yet commercially available.

### 5.3 In vitro keratinocyte dissociation assay

**FIGURE 5:**
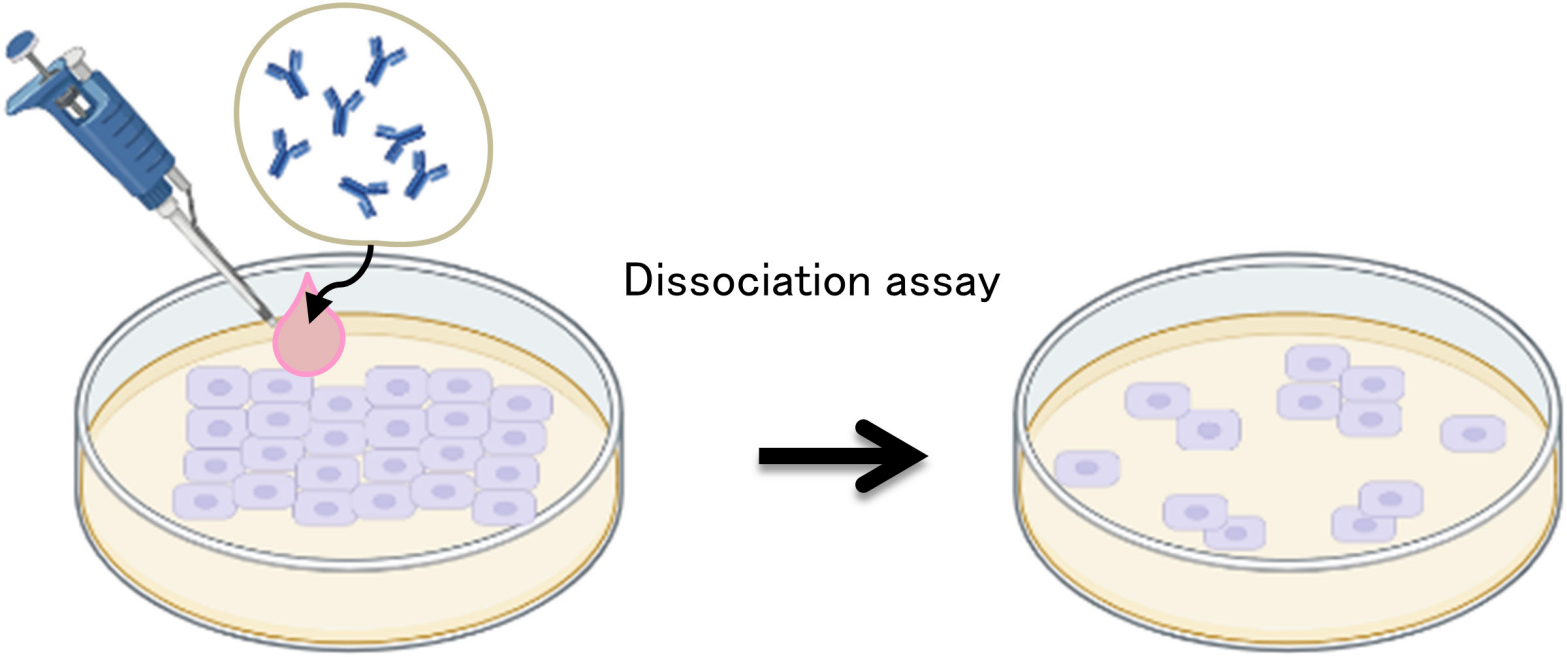
In vitro dissociation assay

The keratinocyte dissociation assay or dispase‐based keratinocyte dissociation assay was originally developed to study cell–cell interactions, and was successfully adapted to the study of pemphigus. The idea behind this protocol is to evaluate the effect of different stimuli on the loss of kerationocyte adhesion in cell monolayers.^51^, ^52^ This method requires the generation of a cell monolayer; administration of different stimuli, such as IgG fractions prepared from pemphigus patients' sera, or monoclonal antibody directed against Dsg3 (i.e., AK23) or other engineered anti‐Dsg3 and/or Dsg1 antibodies (scFv), Dsc3; dispase‐based detachment of cell monolayers from the cell culture dish; application of mechanical stress by standardized pipetting for monolayer fragmentation; and quantification of fragments (Figure 5).

## 6 PATHOGENICITY OF ANTI‐DSC AUTOANTIBODIES

The pathogenic significance of anti‐Dsc autoantibodies in pemphigus is largely unknown, whereas that of anti‐Dsg autoantibodies is well established. Until now, there has been no clear evidence for involvement of anti‐Dscs autoantibodies in pemphigus, but several studies suggesting pathogenicity of anti‐Dscs autoantibodies have been reported. Among Dsc1‐3, the importance of Dsc3 in cell adhesion was confirmed by skin‐specific Dsc3 knockout mice, which showed intraepidermal blistering, resembling that observed in patients with PV.^22^ The capability of anti‐Dsc3 autoantibodies to enforce the loss of keratinocyte adhesion was demonstrated by the keratinocyte dissociation assay, in which the number of fractions of cultured keratinocyte sheets were counted, after incubation with anti‐Dsc3 autoantibodies purified from sera of non‐classical pemphigus.^53^, ^54^

On the other hand, an active disease model of pemphigus expressing anti‐Dsc3 autoantibodies or anti‐Dsc3 and anti‐Dsg3 autoantibodies were developed the pathogenic activity of anti‐Dsc3 autoantibodies. This approach included the adoptive transfer of Dsc3 and/or Dsg3 lymphocytes to Rag2−/− immunodeficient mice that express Dsc3 and Dsg3. The presence of anti‐Dsc3 autoantibodies was sufficient to determine the appearance of a pathological phenotype relatable to pemphigus, but with features not completely superimposable to those observed in the Dsg3 active model, suggesting that the Dsc3 active model might mimic atypical pemphigus. Dsc3 active model was supported the concept that antigens other than Dsgs may be responsible for different phenotypes in pemphigus.^55^ Moreover, when a mixture of splenocytes derived from Dsc3‐immunized animals and Dsg3‐immunized mice were injected in the same Rag2−/− mouse, the presence of both anti‐Dsc3 and anti‐Dsg3 antibodies determines a more severe phenotype and a slower response to prednisolone.

A recent epitope mapping study showed that anti‐Dsc3 autoantibodies in atypical pemphigus patient sera preferentially target the extracellular 2 domain of Dsc3, which is different from the epitope for anti‐Dsg3 autoantibodies in classical pemphigus.^56^ This uniqueness may lead to new approaches to investigate the pathogenicity of pemphigus, particularly with anti‐Dsc autoantibodies, which are different from those used mainly on Dsgs. These findings suggest that, in addition to the steric hindrance mechanism, other pathogenic mechanisms affecting cellular response may also be involved in positive pemphigus with anti‐Dsc3 autoantibodies.

These research results indicated that anti‐Dsc autoantibodies may play a pathogenic role in pemphigus. It may be possible that these cases are diagnosed as anti‐Dsc pemphigus, a new disease entity. Autoantibodies to Dscs, but not to Dsgs, may play a significant role in the pathogenecity of pemphigus, because some cases did not react with Dsgs. Additionally, immunoreactivity with Dscs may explain the unique clinical phenotypes of atypical pemphigus.

## 7 CONCLUSION

Desmocollins is one of the minor autoantigens in pemphigus patients and the autoantibodies against Dsc may be produced by several possible mechanisms. The presence of anti‐Dsc autoantibodies implied several particular clinical features of pemphigus patients, such as intercellular IgA dermatosis, PNP, PH, PVeg, and atypical pemphigus. Currently, a plethora of clinical studies indicate that an autoantibody against Dsc alone could contribute to the pathogenesis of pemphigus and that the anti‐Dsc autoantibodies may interfere with the assembling of the desmosomes in vivo. However, the exact pathogenicity of anti‐Dsc autoantibodies remains unclear. Further studies are required to reveal pathogenicity and clinicopathological relationship of pemphigus with anti‐Dscs autoantibodies. Better understanding of the interaction between Dsc and Dsc‐specific autoantibodies may lead to more targeted therapy for pemphigus patients, especially for pemphigus patients with anti‐Dsc autoantibodies.

## CONFLICT OF INTEREST

No conflict of interest.

## References

[1] Egami S , Yamagami J , Amagai M . Autoimmune bullous skin diseases, pemphigus and pemphigoid. J Allergy Clin Immunol. 2020;145:1031–47. 32272980 10.1016/j.jaci.2020.02.013

[2] Stanley JR , Amagai M . Pemphigus, bullous impetigo, and the staphylococcal scalded‐skin syndrome. N Engl J Med. 2006;355:1800–10. 17065642 10.1056/NEJMra061111

[3] Kasperkiewicz M , Ellebrecht CT , Takahashi H , Yamagami J , Zillikens D , Payne AS , et al. Pemphigus. Nat Rev Dis Primers. 2017;3:17026. 28492232 10.1038/nrdp.2017.26 PMC5901732

[4] Amagai M , Komai A , Hashimoto T , Shirakata Y , Hashimoto K , Yamada T , et al. Usefulness of enzyme‐linked immunosorbent assay using recombinant desmogleins 1 and 3 for serodiagnosis of pemphigus. Br J Dermatol. 1999;140:351–7. 10233237 10.1046/j.1365-2133.1999.02752.x

[5] Hashimoto T , Kiyokawa C , Mori O , Miyasato M , Chidgey MA , Garrod DR , et al. Human desmocollin 1 (Dsc1) is an autoantigen for the subcorneal pustular dermatosis type of IgA pemphigus. J Invest Dermatol. 1997;109:127–31. 9242496 10.1111/1523-1747.ep12319025

[6] Preisz K , Horva'th A , Sa'rdy M , Somlai B , Ha'rsing J , Amagai M , et al. Exacerbation of paraneoplastic pemphigus by cyclophosphamide treatment: detection of novel autoantigens and bronchial autoantibodies. Br J Dermatol. 2004;150:1018–24. 15149520 10.1111/j.1365-2133.2004.05978.x

[7] Saruta H , Ishii N , Teye K , Ono F , Ohyama B , Koga H , et al. Two cases of pemphigus vegetans with IgG anti‐desmocollin 3 antibodies. JAMA Dermatol. 2013;149:1209–13. 23946009 10.1001/jamadermatol.2013.5244

[8] Ueda A , Ishii N , Temporin K , Yamazaki R , Murakami F , Fukuda S , et al. IgA pemphigus with paraneoplastic pemphigus‐like clinical features showing IgA antibodies to desmoglein 1/3 and desmocollin 3, and IgG and IgA antibodies to the basement membrane zone. Clin Exp Dermatol. 2013;38:370–3. 23517469 10.1111/ced.12050

[9] Ueda A , Ishii N , Teye K , Dainichi T , Ohyama B , Hamada T , et al. Unique herpetiform bullous dermatosis with IgG antibodies to desmocollins 1/3 and LAD‐1. Br J Dermatol. 2013;169:719–21. 23607807 10.1111/bjd.12398

[10] Green KJ , Simpson CL . Desmosomes: new perspectives on a classic. J Invest Dermatol. 2007;127:2499–515. 17934502 10.1038/sj.jid.5701015

[11] Garrod D , Chidgey M . Desmosome structure, composition and function. Biochim Biophys Acta. 2008;1778:572–87. 17854763 10.1016/j.bbamem.2007.07.014

[12] Dusek RL , Godsel LM , Green KJ . Discriminating roles of desmosomal cadherins: beyond desmosomal adhesion. J Dermatol Sci. 2007;45:7–21. 17141479 10.1016/j.jdermsci.2006.10.006

[13] Collins JE , Legan PK , Kenny TP , MacGarvie J , Holton JL , Garrod DR . Cloning and sequence analysis of desmosomal glycoproteins 2 and 3 (desmocollins): cadherin‐like desmosomal adhesion molecules with heterogeneous cytoplasmic domains. J Cell Biol. 1991;113:381–91. 2010468 10.1083/jcb.113.2.381 PMC2288940

[14] Troyanovsky SM , Eshkind LG , Troyanovsky RB , Leube RE , Franke WW . Contributions of cytoplasmic domains of desmosomal cadherins to desmosome assembly and intermediate filament anchorage. Cell. 1993;72:561–5. 7679953 10.1016/0092-8674(93)90075-2

[15] Troyanovsky SM , Troyanovsky RB , Eshkind LG , Leube RE , Franke WW . Identification of amino acid sequence motifs in desmocollin, a desmosomal glycoprotein, that are required for plakoglobin binding and plaque formation. Proc Natl Acad Sci. 1994;91:10790–4. 7971964 10.1073/pnas.91.23.10790 PMC45111

[16] Kowalczyk AP , Bornslaeger EA , Borgwardt JE , Palka HL , Dhaliwal AS , Corcoran CM , et al. The amino‐terminal domain of desmoplakin binds to plakoglobin and clusters desmosomal cadherin‐plakoglobin complexes. J Cell Biol. 1997;139:773–84. 9348293 10.1083/jcb.139.3.773 PMC2141713

[17] Hegazy M , Perl AL , Svoboda SA , Green KJ . Desmosomal Cadherins in health and disease. Annu Rev Pathol. 2022;17:47–72. 34425055 10.1146/annurev-pathol-042320-092912 PMC8792335

[18] Chitaev NA , Troyanovsky SM . Direct Ca2+−dependent heterophilic interaction between desmosomal cadherins, desmoglein and desmocollin, contributes to cell‐cell adhesion. J Cell Biol. 1997;138:193–201. 9214392 10.1083/jcb.138.1.193 PMC2139935

[19] Getsios S , Amargo EV , Dusek RL , Ishii K , Sheu L , Godsel LM , et al. Coordinated expression of desmoglein 1 and desmocollin 1 regulates intercellular adhesion. Differentiation. 2004;72:419–33. 15606501 10.1111/j.1432-0436.2004.07208008.x

[20] Marcozzi C , Burdett ID , Buxton RS , Magee AI . Coexpression of both types of desmosomal cadherin and plakoglobin confers strong intercellular adhesion. J Cell Sci. 1998;111:495–509. 9443898 10.1242/jcs.111.4.495

[21] Chidgey M , Brakebusch C , Gustafsson E , Cruchley A , Hail C , Kirk S , et al. Mice lacking desmocollin 1 show epidermal fragility accompanied by barrier defects and abnormal differentiation. J Cell Biol. 2001;155:821–32. 11714727 10.1083/jcb.200105009 PMC2150874

[22] Chen J , Den Z , Koch PJ . Loss of desmocollin 3 in mice leads to epidermal blistering. J Cell Sci. 2008;121:2844–9. 18682494 10.1242/jcs.031518 PMC2659849

[23] Koch PJ , Mahoney MG , Ishikawa H , Pulkkinen L , Uitto J , Shultz L , et al. Targeted disruption of the pemphigus vulgaris antigen (desmoglein 3) gene in mice causes loss of keratinocyte cell adhesion with a phenotype similar to pemphigus vulgaris. J Cell Biol. 1997;137:1091–102. 9166409 10.1083/jcb.137.5.1091 PMC2136216

[24] Mahoney MG , Wang Z , Rothenberger K , Koch PJ , Amagai M , Stanley JR . Explanations for the clinical and microscopic localization of lesions in pemphigus foliaceus and vulgaris. J Clin Invest. 1999;103:461–8. 10021453 10.1172/JCI5252 PMC408100

[25] Stanley JR . Pemphigus. In: Freedberg IM , Eisen AZ , Wolff K , Austen KF , Goldsmith LA , Katz SI , editors. Fitzpatrick's dermatology in general medicine. Volume 1. 6th ed. New York: McGraw‐Hill; 2003. p. 558–67.

[26] Nie Z , Merritt A , Rouhi‐Parkouhi M , Tabernero L , Garrod D . Membrane‐impermeable cross‐linking provides evidence for homophilic, isoform‐specific binding of desmosomal cadherins in epithelial cells. J Biol Chem. 2011;286:2143–54. 21098030 10.1074/jbc.M110.192245 PMC3023511

[27] Harrison OJ , Brasch J , Lasso G , Katsamba PS , Ahlsen G , Honig B , et al. Structural basis of adhesive binding by desmocollins and desmogleins. Proc Natl Acad Sci U S A. 2016;113:7160–5. 27298358 10.1073/pnas.1606272113 PMC4932976

[28] Evangelista F , Roth AJ , Prisayanh P , Temple BR , Li N , Qian Y , et al. Pathogenic IgG4 autoantibodies from endemic pemphigus foliaceus recognize a desmoglein‐1 conformational epitope. J Autoimmun. 2018;89:171–85. 29307589 10.1016/j.jaut.2017.12.017 PMC5902409

[29] Ishii K , Yoshida K , Stanley JR , Yamagami J , Amagai M , Ishiko A . Pemphigus vulgaris and foliaceus IgG autoantibodies directly block heterophilic transinteraction between desmoglein and desmocollin. J Invest Dermatol. 2020;140:1919–26. 32142800 10.1016/j.jid.2020.02.010

[30] Hashimoto T . Immunopathology of IgA pemphigus. Clin Dermatol. 2001;19:683–9. 11705676 10.1016/s0738-081x(00)00193-0

[31] Robinson ND , Hashimoto T , Amagai M , Chan LS . The new pemphigus variants. J Am Acad Dermatol. 1999;40:649–71. 10321591 10.1016/s0190-9622(99)70145-3

[32] Tsuruta D , Ishii N , Hamada T , Ohyama B , Fukuda S , Koga H , et al. IgA pemphigus. Clin Dermatol. 2011;29:437–42. 21679872 10.1016/j.clindermatol.2011.01.014

[33] Ishii N , Ishida‐Ymammoto A , Hashimoto T . Immunolocalization of target autoantigens in IgA pemphigus. Clin Exp Dermatol. 2004;29:62–6. 14723725 10.1111/j.1365-2230.2004.01436.x

[34] Patel PM , Jones VA , Cordova A , Amber KT . IgA pemphigus: a systematic review. J Am Acad Dermatol. 2020;82:1386–92. 31812619 10.1016/j.jaad.2019.11.059

[35] Wallach D . Intraepidermal IgA pustulosis. J Am Acad Dermatol. 1992;27:993–1000. 1479108 10.1016/0190-9622(92)70301-u

[36] Stendahl O , Molin L , Dahlgren C . The inhibition of polymorphonuclear leoukocyte cytotoxicity by dapsone: a possible mechanism in the treatment of dermatitis herpetiformis. J Clin Invest. 1978;62:214–22. 207742 10.1172/JCI109109 PMC371756

[37] Iranzo P , Ishii N , Hashimoto T , Alsina‐Gibert M . Nonclassical pemphigus with exclusively IgG anti‐desmocollin 3‐specific antibodies. Australas J Dermatol. 2019;60:e217–9. 30671942 10.1111/ajd.12991

[38] Anhalt GJ , Kim SC , Stanley JR , Korman NJ , Jabs DA , Kory M , et al. Paraneoplastic pemphigus. An autoimmune mucocutaneous disease associated with neoplasia. N Engl J Med. 1990;323:1729–35. 2247105 10.1056/NEJM199012203232503

[39] Kim SC , Kwon YD , Lee IJ , Chang SN , Lee TG . cDNA cloning of the 210‐kDa paraneoplastic pemphigus antigen reveals that envoplakin is a component of the antigen complex. J Invest Dermatol. 1997;109:365–9. 9284106 10.1111/1523-1747.ep12336235

[40] Oursler JR , Labib RS , Ariss‐Abdo L , Burke T , O'Keefe EJ , Anhalt GJ . Human autoantibodies against desmoplakins in paraneoplastic pemphigus. J Clin Invest. 1992;89:1775–82. 1601988 10.1172/JCI115781 PMC295873

[41] Kiyokawa C , Ruhrberg C , Nie Z , Karashima T , Mori O , Nishikawa T , et al. Envoplakin and periplakin are components of the paraneoplastic pemphigus antigen complex. J Invest Dermatol. 1998;111:1236–8. 9856851 10.1046/j.1523-1747.1998.00449.x

[42] Schepens I , Jaunin F , Begre N , Läderach U , Marcus K , Hashimoto T , et al. The protease inhibitor alpha‐2‐macroglobulin‐like‐1 is the p170 antigen recognized by paraneoplastic pemphigus autoantibodies in human. PLoS One. 2010;5:e12250. 20805888 10.1371/journal.pone.0012250 PMC2923615

[43] Schmidt E , Kasperkiewicz M , Joly P . Pemphigus. Lancet. 2019;394:882–94. 31498102 10.1016/S0140-6736(19)31778-7

[44] Ishii N , Teye K , Fukuda S , Uehara R , Hachiya T , Koga H , et al. Anti‐desmocollin autoantibodies in nonclassical pemphigus. Br J Dermatol. 2015;173:59–68. 25640111 10.1111/bjd.13711

[45] Jablonska S , Chorzelski TP , Beutner EH , Jarzabek‐Chorzelska M . Herpetiform pemphigus, a variable pattern of pemphigus. Int J Dermatol. 1975;14:353–9. 1097347 10.1111/j.1365-4362.1975.tb00125.x

[46] Costa LMC , Cappel MA , Keeling JH . Clinical, pathologic, and immunologic features of pemphigus herpetiformis: a literature review and proposed diagnostic criteria. Int J Dermatol. 2019;58:997–1007. 30900757 10.1111/ijd.14395

[47] Ohata Y , Komiya H , Kawahara Y , Watanabe K , Nishikawa T , Hashimoto T . A case of Newmann type pemphigus vegetans showing reactivity with the 130 kD pemphigus vulgaris antigen. Acta Derm Venereol. 1996;76:169–70. 8740287 10.2340/0001555576169170

[48] Hashimoto K , Hashimoto T , Higashiyama M , Nishikawa T , Garrod DR , Yoshikawa K . Detection of anti‐desmocollins I and II autoantibodies in two cases of Hallopeau type pemphigus vegetans by immunoblot analysis. J Dermatol Sci. 1994;7:100–6. 8060912 10.1016/0923-1811(94)90083-3

[49] Hisamatsu Y , Amagai M , Garrod DR , Kanzaki T , Hashimoto T . The detection of IgG and IgA autoantibodies to desmocollins 1‐3 by enzyme‐linked immunosorbent assays using baculovirus‐expressed proteins, in atypical pemphigus but not in typical pemphigus. Br J Dermatol. 2004;151:73–83. 15270874 10.1111/j.1365-2133.2004.05995.x

[50] Teye K , Numata S , Ohzono A , Ohyama B , Tsuchisaka A , Koga H , et al. Establishment of IgA ELISAs of mammalian recombinant proteins of human desmocollins 1‐3. J Dermatol Sci. 2016;83:75–7. 27150020 10.1016/j.jdermsci.2016.04.001

[51] Calautti V , Cabodi S , Stein PL , Hatzfeld M , Kedersha N , Dotto GP . Tyrosine phosphorylation and Src family kinases control keratinocyte cell‐cell adhesion. J Cell Biol. 1998;141:1449–65. 9628900 10.1083/jcb.141.6.1449 PMC2132783

[52] Caldelari R , de Bruin A , Baumann D , Suter MM , Bierkamp C , Balmer V , et al. A central role for the armadillo protein Plakoglobin in the autoimmune disease pemphigus vulgaris. J Cell Biol. 2001;153:823–34. 11352942 10.1083/jcb.153.4.823 PMC2192383

[53] Mao X , Nagler AR , Farber SA , Choi EJ , Jackson LH , Leiferman KM , et al. Autoimmunity to desmocollin 3 in pemphigus vulgaris. Am J Pathol. 2010;177:2724–30. 20952584 10.2353/ajpath.2010.100483 PMC2993297

[54] Rafei D , Müller R , Ishii N , Llamazares M , Hashimoto T , Hertl M , et al. IgG autoantibodies against desmocollin 3 in pemphigus sera induce loss of keratinocyte adhesion. Am J Pathol. 2011;178:718–23. 21281804 10.1016/j.ajpath.2010.10.016 PMC3069870

[55] Lotti R , Atene CG , Marconi A , Di Rocco G , Reggiani Bonetti L , Zanocco Marani T , et al. Development of a desmocollin‐3 active mouse model recapitulating human atypical pemphigus. Front Immunol. 2019;10:1387. 31275323 10.3389/fimmu.2019.01387 PMC6593104

[56] Koga H , Teye K , Otsuji Y , Ishii N , Hashimoto T , Nakama T . Autoantibodies to DSC3 in pemphigus exclusively recognize calcium‐dependent epitope in extracellular domain 2. J Invest Dermatol. 2021;141:2123–31.e2. 33766509 10.1016/j.jid.2021.01.032

